# Individuals carrying the HLA-B*15 allele exhibit favorable responses to COVID-19 vaccines but are more susceptible to Omicron BA.5.2 and XBB.1.16 infection

**DOI:** 10.3389/fimmu.2024.1440819

**Published:** 2024-08-27

**Authors:** Lingxin Meng, Yue Pan, Yueping Liu, Rui He, Yuting Sun, Chenhui Wang, Lei Fei, Airu Zhu, Zhongfang Wang, Yunfei An, Yuzhang Wu, Bo Diao, Yongwen Chen

**Affiliations:** ^1^ Institute of Immunology, PLA, Third Military Medical University, Chongqing, China; ^2^ Department of Medical Laboratory Center, General Hospital of Central Theater Command, Wuhan, Hubei, China; ^3^ Beijing Institute of Microbiology and Epidemiology, Beijing, China; ^4^ State Key Laboratory of Respiratory Disease & National Clinical Research Center for Respiratory Disease, Guangzhou Institute of Respiratory Health, The First Affiliated Hospital of Guangzhou Medical University, Guangzhou Medical University, Guangzhou, China; ^5^ Department of Rheumatology and Immunology, Children’s Hospital of Chongqing Medical University, Chongqing, China

**Keywords:** COVID-19, Omicron variants, HLA-B*15, vaccination, SARS-CoV-2

## Abstract

**Background:**

Natural infection or vaccination have provided robust immune defense against SARS-CoV-2 invasion, nevertheless, Omicron variants still successfully cause breakthrough infection, and the underlying mechanisms are poorly understood.

**Methods:**

Sequential blood samples were continuously collected at different time points from 252 volunteers who were received the CanSino Ad5-nCoV (n= 183) vaccine or the Sinovac CoronaVac inactivated vaccine (n= 69). The anti-SARS-CoV-2 prototype and Omicron BA.5.2 as well as XBB.1.16 variant neutralizing antibodies (Nab) in sera were detected by ELISA. Sera were also used to measure pseudo and live virus neutralization assay. The associations between the anti-prototype Nab levels and different HLA-ABC alleles were analyzed using artificial intelligence (AI)-deep learning techniques. The frequency of B cells in PBMCs was investigated by flow cytometry assay (FACs).

**Results:**

Individuals carrying the HLA-B^*^15 allele manifested the highest concentrations of anti-SARS-CoV-2 prototype Nab after vax administration. Unfortunately, these volunteers are more susceptible to Omicron BA.5.2 breakthrough infection due to their sera have poorer anti-BA.5.2 Nab and lower levels of viral neutralization efficacy. FACs confirmed that a significant decrease in CD19^+^CD27^+^RBD^+^ memory B cells in these HLA-B^*^15 population compared to other cohorts. Importantly, generating lower concentrations of cross-reactive anti-XBB.1.16 Nab post-BA.5.2 infection caused HLA-B^*^15 individuals to be further infected by XBB.1.16 variant.

**Conclusions:**

Individuals carrying the HLA-B^*^15 allele respond better to COVID-19 vax including the CanSino Ad5-nCoV and the Sinovac CoronaVac inactivated vaccines, but are more susceptible to Omicron variant infection, thus, a novel vaccine against this population is necessary for COVID-19 pandemic control in the future.

Since its initial emergence in Wuhan at the end of 2019, Severe Acute Respiratory Syndrome Coronavirus 2 (SARS-CoV-2) has caused more than 778 million confirmed cases of coronavirus disease 2019 (COVID-19), and more than 6.3 million fatalities worldwide as of February 2024 (1). SARS-CoV-2 has undergone continuous genetic evolution, and multiple mutation variants including Omicron have been reported (2, 3). Over 248 COVID-19 vaccines have been developed globally, and 9 have emergency been used authorization (4, 5). These vaccines have been reported to successfully induce protective humoral and T-cell-mediated immunity against SARS-CoV-2. For instance, individuals vaccinated with the Ad26.COV2.S or the Pfizer BNT162b2 presented robust neutralizing antibodies (Nab) and strong T-cell responses against the prototype strain, Delta, and Omicron variants (6). Additionally, SARS-CoV-2 spike-specific CD4^+^ and CD8^+^ T cells induced by BNT162b2 provide extensive immune coverage against Omicron B.1.1.529 variant (7). However, Omicron variants can still penetrate vaccine induced defenses and cause breakthrough infection, but the mechanisms underlying immune evasion remain unclear.

Human leukocyte antigen (HLA) has been reported to be related to COVID-19 vaccination efficacy, and certain HLA alleles were showed to increase susceptibility to SARS-CoV-2 infection (8, 9). For examples, people carrying the HLA-DQA1^*^03:03, DRB1^*^12:01, DRB1^*^03:01, or the HLA-DRB1^*^07:01, exhibited high levels of serum Nab after received BNT162b2 vaccine (10, 11). Populations with the HLA-DQB1^*^06 allele had markedly increased in Nab levels post ChAdOx1, BNT162b2, or mRNA-1273 vaccinations. Conversely, some studies reported that certain HLA supertypes are negatively correlated with vaccine efficacy (12). Unfortunately, no data are available about the associations between certain HLA allele and the levels of anti-SARS-CoV-2 prototype Nab induced by the Ad5-nCoV or CoronaVac inactivated vaccines till now.

Here, 252 volunteers received the Ad5-nCoV (CanSino, n = 183) or the CoronaVac inactivated vaccine (Sinovac, n = 69) were recruited, and sequential blood samples were collected at different time points. We showed that individuals carrying the HLA-B^*^15 allele have a good Nab response to COVID-19 vaccines, unfortunately, these populations are more susceptible to suffer from Omicron BA.5.2 and XBB.1.16 variant breakthrough infection later.

## 1 Results

### 1.1 Volunteers and sample collection

**Figure 1:**
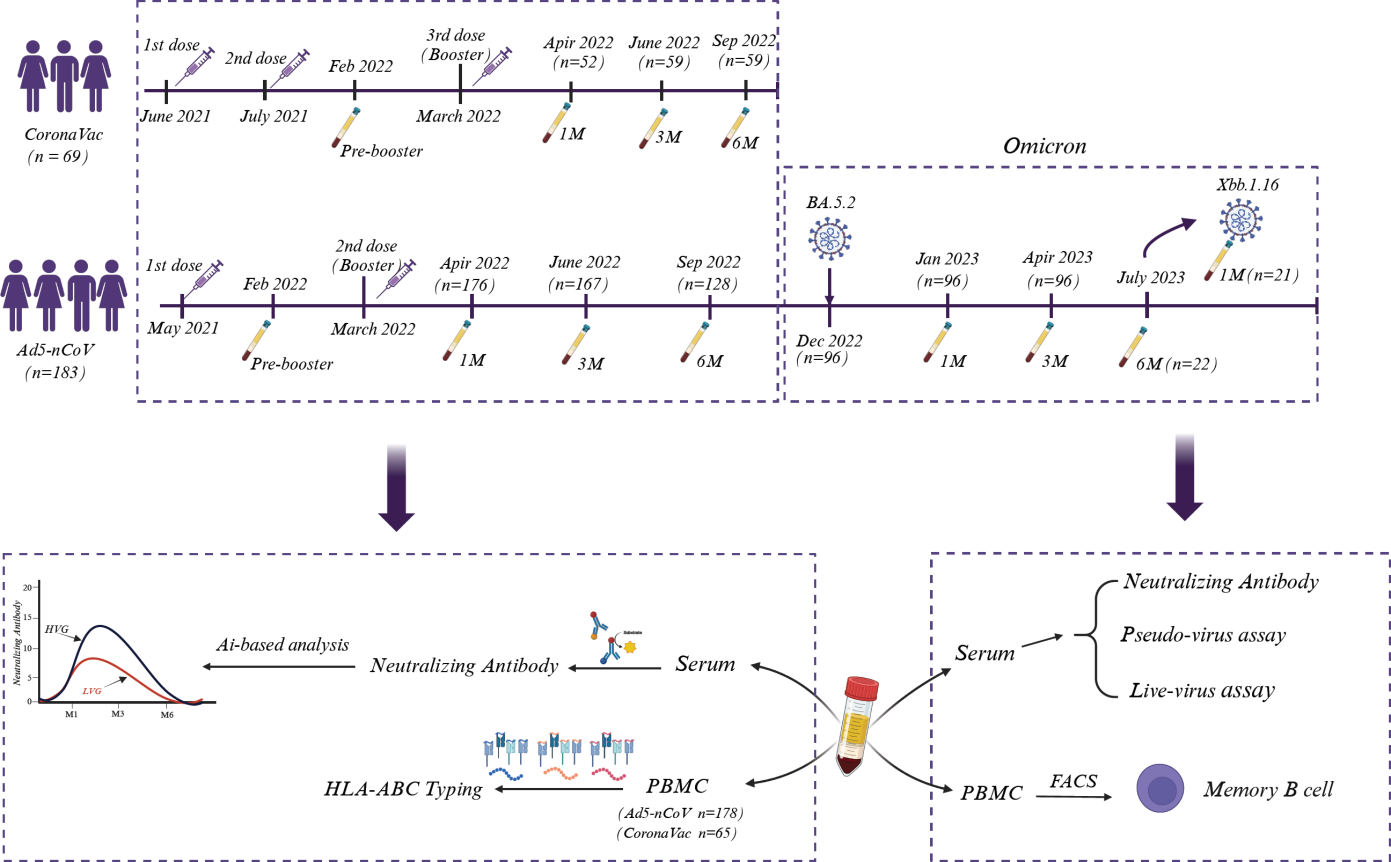
Sampling and experimental setup. A total of 252 volunteers who were administered Ad5-nCoV (CanSino, n = 183) or the CoronaVac inactivated vaccine (Sinovac, n = 69) were recruited, and blood samples were systematically collected on the indicated days. The sera were used to analyze Nab, while PBMCs were selected for HLA-ABC typing, and the associations between Nab levels and HLA-ABC alleles were analyzed via AI-based analysis. Moreover, sera from some Ad5-nCoV-treated volunteers were also used to detect the efficacy of liver and pseudovirus neutralization. Fluorescence-activated cell sorting (FACs) was used to identify SARS-CoV-2-specific memory B cells.

A total of 252 volunteers were recruited for this study, 183 participants (149 males and 34 females) were inoculated with the Ad5-nCoV vaccine (CanSino, China), and 69 volunteers (38 males and 31 females) were administered by the CoronaVac inactivated vaccine (Sinovac, China). In addition, we acquired 10 normal plasma samples from the First Affiliated Hospital of Guangzhou Medical University before the COVID-19 pandemic. Detail information of individuals vaccinated with both vaccines were showed in **Figure 1**.

We continuously monitored volunteers who were inoculated with the CanSino Ad5-nCoV vaccine after COVID-19 restrictions were fully lifted in China (December 5, 2022). Some of these volunteers subsequently suffered from Omicron BA.5.2 and XBB.1.16 infection, as confirmed to be positive for SARS-CoV-2 by RT-PCR, and they presented either asymptomatic or mild symptoms (**Supplementary Tables 1**, **2**). Sera and peripheral blood mononuclear cells (PBMCs) were separated by centrifugation. Serum samples were used to detect SARS-CoV-2-specific antibody, and PBMCs were used for HLA-ABC genotyping. The associations between anti-SARS-CoV-2 prototype Nab levels and different HLA-ABC alleles were further analyzed utilizing artificial intelligence (AI)-deep learning techniques. The efficacy of virus neutralization and the frequency of memory B-cell were also investigated (**Figure 1**).

### 1.2 Volunteers carrying HLA-B^*^15 alleles have a favorable response to COVID-19 vaccines

All volunteers were recruited to measure HLA-ABC genotype polymorphisms using the MiSeqDx™ Next Generation Sequencing (NGS) platform. The predominant alleles of HLA-A supertype in these populations were HLA-A^*^11 (25.63%), HLA-A^*^02 (27.74%), and HLA-A^*^24 (18.7%). For the HLA-B supertype, the leading genotypes were HLA-B^*^40 (16.39%), HLA-B^*^15 (13.24%) and HLA-B^*^46 (9.45%). Among the HLA-C supertype, HLA-C^*^03 (20.8%), HLA-C^*^01 (16.81%), HLA-C^*^07 (11.97%) and HLA-C^*^14 (11.76%) are the principal alleles (**Supplementary Figure 1**).

**Figure 2:**
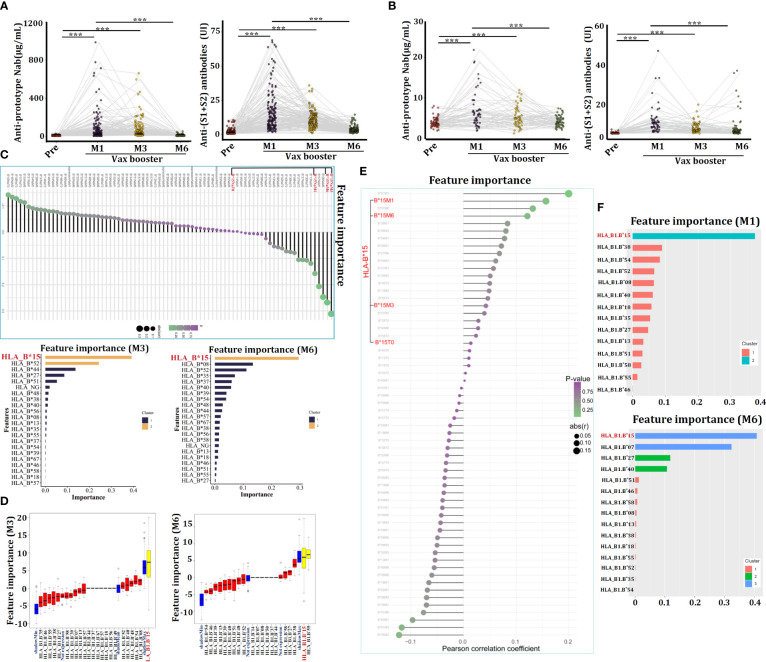
Individuals carrying HLA-B^*^15 alleles exhibit a favorable response to COVID-19 vaccination. **(A)** Serum concentrations of anti-prototype Nab and anti-(S1+S2) antibodies in volunteers who received the CanSino Ad5-nCoV vaccine **(A)** or the Sinovac CoronaVac inactivated vaccine **(B)** at the indicated time points. The relationships between serum anti-prototype Nab concentrations and HLA-ABC alleles in CanSino Ad5-nCoV-vaccinated volunteers were investigated by XGBoost **(C)** and Boruta **(D)**. The associations between serum anti-prototype Nab concentrations and HLA-ABC alleles in volunteers administered the Sinovac CoronaVac inactivated vaccine were investigated by XGBoost **(E, F)**. ***p <0.0001.

The concentrations of anti-SARS-CoV-2-specific prototype Nab and anti-Spike (S1+S2) antibodies in sera were measured by ELISA. For the CanSino Ad5-nCoV vaccine, both types of antibodies were nearly undetectable before vax booster (over 12 months after the initial 1^st^ dose), this result resembled to previous reports (13, 14). However, the concentrations of both antibodies were significantly triggered by the 2^nd^ dose (vax booster) at 1 month (M1), they were sustained for up to 3 months (M3) and gradually vanished after 6 months (M6) (**Figure 2A**). Similar results were also observed in volunteers who were received the Sinovac CoronaVac inactivated vaccine (**Figure 2B**). Therefore, the volunteers were classified into two groups, the high-vax response group (HVG) and low-vax response group (LVG) based on serum concentrations of the anti-prototype Nab (**Supplementary Figure 2**).

The heterogeneity of HLA molecules has been reported to affect the efficacy of COVID-19 vaccines (15). We therefore analyzed the associations between certain HLA-ABC allele and anti-SARS-CoV-2 prototype Nab levels utilizing an AI-driven deep learning technique. Interestingly, XGBoost showed individuals carrying the HLA-B^*^15 allele exhibited the most pronounced increase in anti-prototype Nab levels after vax booster, especially at M3 and M6 compared to Nab in sera of pre-booster (**Figure 2C**). Boruta analysis also confirmed that HLA-B^*^15 persons presented an augmenting anti-prototype Nab (**Figure 2D**). In the context of the Sinovac CoronaVac inactivated vaccine, XGBoost analysis showed that individuals carrying HLA-B^*^15 alleles manifested the highest levels of anti-SARS-CoV-2 Nab after the 3^rd^ dose of vaccine administration, as compared to that in serum from pre-booster (T0) (**Figures 2E, F**), confirming individuals carrying HLA-B^*^15 allele have favorable responses to both the Ad5-nCoV and CoronaVac vaccines.

### 1.3 HLA-B^*^15 volunteers manifested worse anti-SARS-CoV-2 prototype Nab after BA.5.2 infection

**Figure 3:**
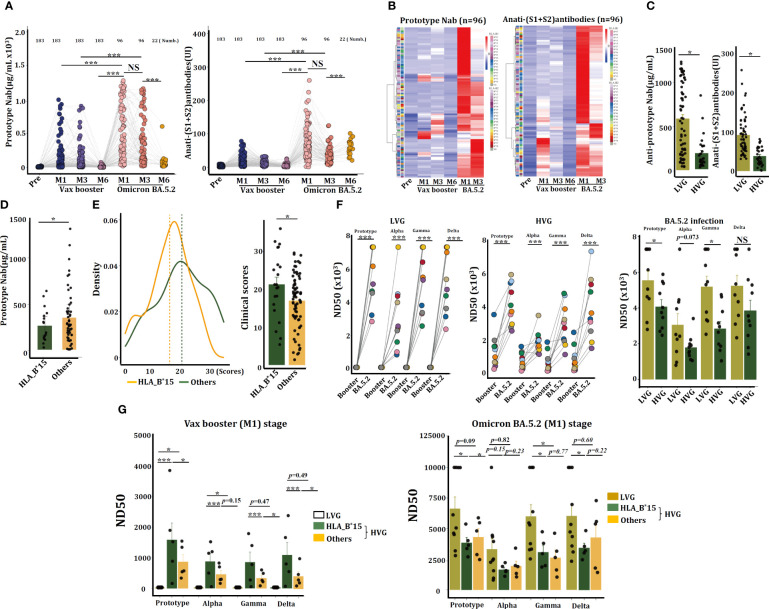
HLA-B^*^15 volunteers exhibited a significant reduction in Nab and viral neutralization efficacy against the SARS-CoV-2 prototype strain after BA.5.2 infection. **(A)** Serum concentrations of anti-prototype Nab and anti-(S1+S2) antibodies at the indicated time points in volunteers who received the CanSino Ad5-nCoV vaccine following BA.5.2 infection. **(B)** Heatmap showing the serum concentrations of anti-prototype Nab and anti-(S1+S2) antibodies at different time points in 96 individuals. **(C)** Serum concentrations of the anti-prototype Nab at M1 post-Omicron BA.5.2 infection were compared between the LVG and HVG groups. **(D)** The volunteers (n=96) were divided into two groups (HLA-B*15 and others), and the serum concentrations of the anti-prototype Nab at M1 after Omicron BA.5.2 infection were compared. **(E)** The scores of clinical symptoms were calculated (left) and compared (right) between HLA-B*15 volunteers and others post-BA.5.2 infection. **(F)** Sera from M1 after the vax booster or post-BA.5.2 infection of the LVG (left) and HVG (right) were used to detect the efficacy of viral neutralization. **(G)** The volunteers were divided into two groups, HLA-B*15 volunteers and others, and the efficacy of viral neutralization of sera was detected and compared. *p <0.05, ** *p <*0.01, and ***p <0.001.

Volunteers received the Ad5-nCoV vaccine presented better the SARS-CoV-2-specific anti-prototype Nab and the anti-(S1+S2) antibodies than that from persons administered with the CoronaVac vaccine (**Supplementary Figure 3**), therefore, individuals received the Ad5-nCoV vaccine were continued to monitor their susceptibility to SARS-CoV-2 infection thereafter. COVID-19 restrictions were fully lifted by Chinese government on December 5^th^, 2022. The timeframe exceeded 9 months after they were received the 2^nd^ dose of Ad5-nCoV vaccine. Unfortunately, all these volunteers were infected by Omicron BA.5.2, as confirmed via SARS-CoV-2-specific RT-PCR. Plasma samples were collected from these individuals after 1 month (M1, n = 96), 3 months (M3, n = 96), and 6 months (M6, n = 22) post infection. ELISA analysis revealed that the anti-prototype Nab and the anti-(S1+S2) antibody levels were increased significantly after BA.5.2 infection, and their concentrations were significantly higher than that triggered by the Ad5-nCoV vaccine (**Figure 3A**).

A heatmap revealed that individuals who exhibited stronger antibody levels caused by Ad5-nCoV vaccine conversely presented with lower antibody concentrations following BA.5.2 infection (**Figure 3B**). Indeed, serum from HVG patients had notably lower levels of both types of antibodies at M1 post-BA.5.2 infection than serum from LVG patients (**Figure 3C**). Individuals with HLA-B^*^15 allele also manifested significantly lower concentrations of the anti-SARS-CoV-2 prototype Nab at M1 post BA.5.2 infection than that from non-HLA-B^*^15 patients (**Figure 3D**). Additionally, the HLA-B^*^15 patients experienced notably more severe clinical manifestations than that in the non-HLA-B^*^15 persons (**Figure 3E**).

The sera from volunteers in the LVG (n = 10) and HVG (n = 10) groups were randomly selected, and the efficacy of viral neutralization was analyzed by a pseudovirus neutralization assay. Results showed that sera from LVG at 1 month of Vax booster (booster-M1) had not any capacity to neutralize SARS-CoV-2 prototype or the close variants, including Alpha, Beta, and Gamma strains, however, serum samples at 1 month of Omicron BA.5.2 infection (BA.5.2-M1) can efficiently neutralize these viruses. Sera from HVG group of booster-M1 have the capacity to neutralize SARS-CoV-2, and samples at BA.5.2-M1 further enhanced the efficacy. Interestingly, sera from the LVG cohort at BA.5.2-M1 exhibited dramatically greater viral neutralization efficacy than those from the HVG cohort (**Figure 3F**). Individuals in the HVG cohort were further divided into HLA-B^*^15 and non-HLA-B^*^15 (others) groups. In the Vax booster-M1 stage, it seems that serum from HLA-B^*^15 individuals displayed better viral neutralization efficacy than that from LVG individuals or non-HLA-B^*^15 (others), however, this effect was oppositely observed after BA.5.2 infection (BA.5.2-M1), and the neutralization efficiency of HLA-B^*^15 sera was reduced dramatically against the SARS-CoV-2 prototype strain and Gamma and Delta variants (**Figure 3G**), suggesting that sera from HLA-B^*^15 volunteers diminished viral neutralization efficacy after BA.5.2 infection.

### 1.4 HLA-B^*^15 populations have worse anti-BA.5.2-specific Nab post BA.5.2 infection

**Figure 4:**
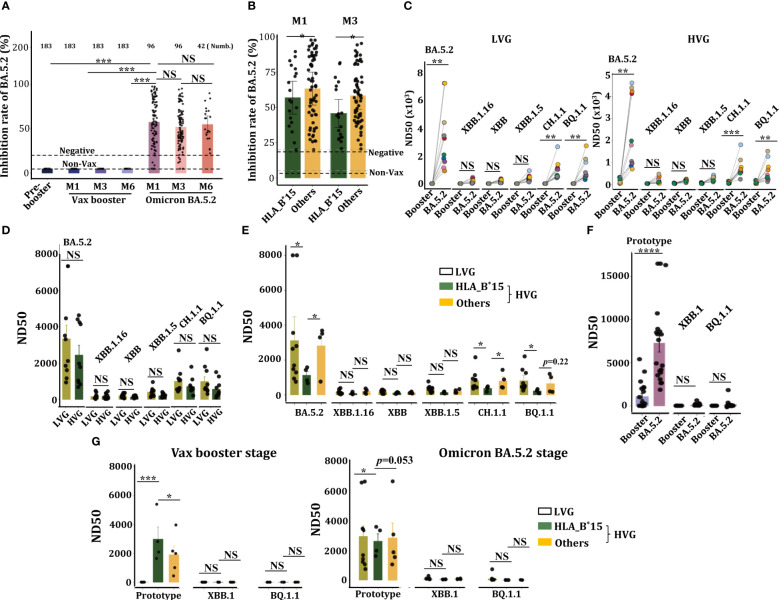
HLA-B^*^15-treated volunteers exhibited a significant reduction in Nab and viral neutralization efficacy against Omicron BA.5.2 post infection. **(A)** Serum concentrations of anti-BA.5.2 Nab at the indicated time points in volunteers who received the CanSino Ad5-nCoV vaccine following BA.5.2 infection. **(B)** The volunteers were divided into two groups, HLA-B*15 volunteers and others, and the serum concentrations of anti-BA.5.2 Nab at M1 and M3 after Omicron BA.5.2 infection were compared between HLA-B^*^15 volunteers and others. **(C)** Sera from M1 after the vax booster or post-BA.5.2 infection of LVG (left) and HVG (right) were used to detect the efficacy of viral neutralization against Omicron variants. **(D)** The efficacy of viral neutralization against Omicron variants in sera collected at M1 after Omicron BA.5.2 infection was compared between the LVG and HVG groups. **(E)** Individuals from the HVG group were further divided into HLA-B*15 volunteers and others, and the efficacy of viral neutralization against Omicron variants from sera collected at M1 after Omicron BA.5.2 infection was compared. **(F)** The efficacy of live viral neutralization from sera collected at M1 after vax booster and post-Omicron BA.5.2 infection. **(G)** The efficacy of live viral neutralization from sera collected at M1 after vax booster (left) and post-Omicron BA.5.2 infection (right) was compared among the LVG and HLA-B*15 alleles and others. NS: not significantly different, *p <0.05, ** *p <*0.01, and ***p <0.001.

The concentrations of anti-BA.5.2 specific neutralization antibodies (anti-BA.5.2 Nab) in these continuous samples were measured, results showed that Ad5-nCoV vaccine has not the capacity to trigger anti-BA.5.2 Nab (inhibition rate of BA.5.2) production (**Figure 4A**), indicating Ad5-nCoV vaccine failed to induce a cross-protective humoral response against BA.5.2 variant. However, high levels of anti-BA.5.2 specific Nab were observed at both M1 and M3 following BA.5.2 infection, and their concentrations were sustained for 6 months at least (**Figure 4A**). Surprisingly, the serum levels of anti-BA.5.2 Nab from HLA-B^*^15 individuals were significantly lower than that from non-HLA-B^*^15 (others) cohorts (**Figure 4B**), confirming that HLA-B^*^15 individuals had worse humoral responses post BA.5.2 infection.

We then analyzed the efficacy of virus neutralization of these serum samples. Sera from both LVG and HVG groups at BA.5.2-M1, rather than that from booster-M1, efficiently neutralized Omicron BA.5.2, and they also manifested a slight neutralization efficacy against BQ.1.1 and CH.1.1, however, no neutralization efficacy was observed against XBB, XBB.1.16 or XBB.1.1 variants (**Figure 4C**). Anticipated, sera from LVG and HVG at BA.5.2-M1 stage also manifested high efficacy of virus neutralization (**Figure 4D**), notably, serum from HLA-B^*^15 individuals showed a markedly reduced in viral neutralization capacity against Omicron BA.5.2, BQ.1.1 and CH.1.1 variants, compared to that of individuals in both the LVG and HVG groups (**Figure 4E**).

We also analyzed the efficacy of live virus neutralization of these sera. Expectedly, sera at both the booster-M1 and the BA.5.2-M1 stage have the capacity to neutralize SARS-CoV-2 prototype strain, however, these sera manifested not any neutralization capacity to Omicron XBB.1 or BQ.1.1 variants, and the sera from BA.5.2-M1 manifested greater viral neutralization efficacy than the booster-M1 does (**Figure 4F**). Interestingly, sera from HLA-B^*^15 individuals at the booster-M1 exhibited greater neutralizing activity against SARS-CoV-2 prototype than that from the LVG group or other individuals in the HVG group, conversely, sera from HLA-B^*^15 individuals at the BA.5.2-M1 exhibited a diminished efficacy of virus neutralization (**Figure 4G**), suggesting HLA-B^*^15 individuals exhibited a significant decrease in virus neutralization capacity.

### 1.5 CD19^+^CD27^+^RBD^+^ memory B cells in HLA-B*15 individuals were declined

**Figure 5:**
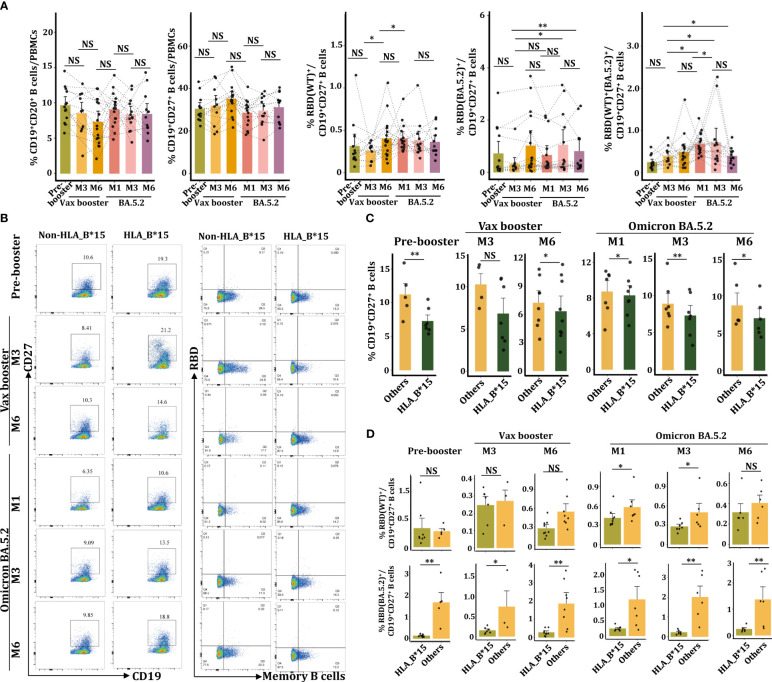
Reduced CD19^+^CD27^+^RBD^+^ memory B cells in the HLA-B*15 population after BA.5.2 infection. **(A)** FACs analysis of the percentages of CD19^+^IgG^+^ B cells, CD19^+^27^+^ memory B cells, CD19^+^27^+^RBD(WT)^+^, CD19^+^27^+^RBD(BA.5.2)^+^, and CD19^+^27^+^RBD(WT)^+^(BA.5.2)^+^ memory B cells at various time points. **(B)** FACs analysis of CD19^+^CD20^+^ B cells and RBD (WT)^+^ memory B cells between the HLA-B*15 and the non-HLA-B*15 groups. **(C)** The percentages of CD19^+^27^+^ memory B cells in HLA-B*15 individuals and other individuals at different time points were compared. **(D)** The percentages of CD19^+^27^+^RBD(WT)^+^ and CD19^+^27^+^RBD(BA.5.2)^+^ memory B cells in HLA-B*15 individuals and other individuals at different time points were compared. NS: not significantly different, *p <0.05, ** *p <*0.01, and ***p <0.001.

Given the critical role of B cells in antibody production, we then examined the status of B cells by FACs. Data from sequential samples (n=10) showed there was no significant difference in the frequency of CD19^+^CD20^+^ B cells or CD19^+^CD27^+^ memory B cells throughout the whole investigated period (**Figure 5A**). Individuals were further divided into HLA-B^*^15 and non-HLA-B^*^15 cohorts (**Figure 5B**). Statistical analysis revealed that HLA-B^*^15 populations had significantly lower percentages of memory B cells than non-HLA-B^*^15 cohorts (others) from the vax booster stage to the Omicron BA.5.2 infection stage (**Figure 5C**).

We further detected the percentage of RBD(WT)^+^ and RBD(BA.5.2)^+^ memory B cells utilizing the recombinant SARS-CoV-2 BA.5.2 RBD Alexa Fluor^®^ 488 protein and the recombinant SARS-CoV-2 spike RBD Alexa Fluor^®^ 647 protein (**Figure 5B**, **Supplementary Figure 4**). As expected, the Vax booster enhanced the number of RBD(WT)^+^CD19^+^CD27^+^ memory B cells, while BA.5.2 infection did not further increase cell frequencies (**Figure 5A**). Compared to vax booster, BA.5.2 infection promoted the percentage of RBD(BA.5.2)^+^CD19^+^CD27^+^ memory B cells (**Figure 5A**). Importantly, the frequency of RBD(WT)^+^(BA.5.2)^+^CD19^+^CD27^+^ memory B cells was dramatically increased following BA.5.2 infection (**Figure 5A**). However, at the booster-M6 and the BA.5.2-M1 as well as the BA.5.2-M3, HLA-B^*^15 individuals manifested dramatically lower numbers of RBD(WT)^+^CD19^+^CD27^+^ memory B cells in PBMCs than that from non-HLA-B^*^15 individuals (**Figure 5D**). Similar results were also observed in the percentage of RBD(BA.5.2)^+^CD19^+^CD27^+^ memory B cells (**Figure 5D**), confirming the reduction of memory B cells in HLA-B^*^15 individuals after BA.5.2 infection.

### 1.6 HLA-B^*^15 individuals are more susceptibility to secondary Omicron XBB.1.16 infection

**Figure 6:**
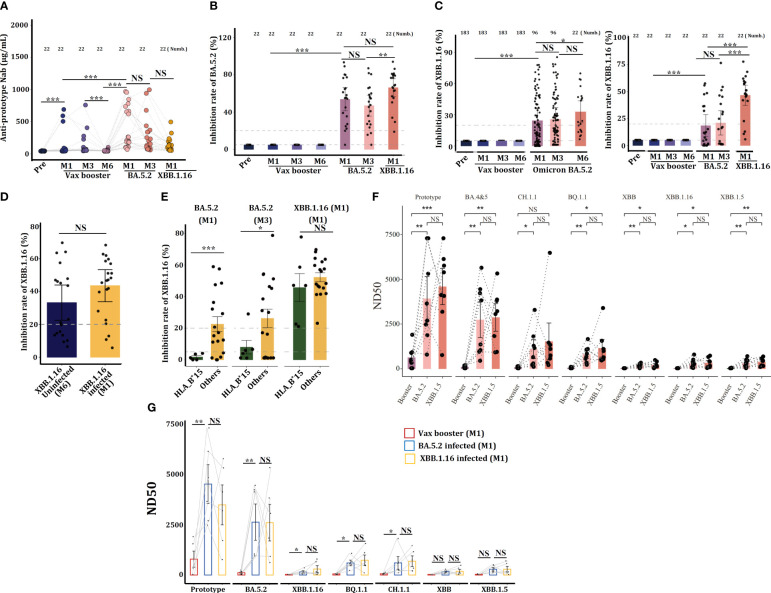
Individuals carrying the HLA-B*15 allele are more susceptible to Omicron XBB.1.16 infections. Serum concentrations of anti-prototype Nab **(A)** and anti-BA.5.2 Nab **(B)** from 21 volunteers who received the CanSino Ad5-nCoV vaccine following infection with Omicron BA.5.2 or XBB.1.16 were detected by ELISA. **(C)** Serum concentrations of anti-XBB.1.16 in Nab volunteers who received the CanSino Ad5-nCoV vaccine following infection with Omicron BA.5.2 (left) and 22 volunteers who were further infected with XBB.1.16 (right) were detected by ELISA. **(D)** Serum concentrations of anti-XBB.1.16 Nab in individuals infected with or without XBB.1.16 were compared. **(E)** Serum concentrations of anti-XBB.1.16 Nab at the indicated time points were compared between HLA-B^*^15 and other antibodies. **(F)** Some volunteers, and **(G)** HLA-B^*^15 sera were collected at M1 after the vax booster and post-Omicron BA.5.2 and XBB.1.16 infection, and the efficacy of viral neutralization was detected. NS: not significantly different, *p <0.05, ** *p <*0.01, and ***p <0.001.

In July 2023, a resurgence of COVID-19 occurred in Chongqing city, and serum samples were collected from 43 volunteers who previously received the Ad5-nCoV vaccine. Based on SARS-CoV-2 antigen detection and symptom assessment, 21 individuals were confirmed to suffer from XBB.1.16 variant infection. ELISA showed that XBB.1.16 infection could not further enhance anti-SARS-CoV-2 Nab levels (**Figure 6A**), however, XBB.1.16 infection increased the concentration of cross-reactive anti-BA.5.2-Nab (**Figure 6B**), suggesting that XBB.1.16 infection enhanced the cross-reactive humoral response to BA.5.2 strain.

Data from continuous samples (n=21) showed that XBB.1.16 infection further enhanced anti-XBB.1.16-specific Nab (**Figure 6C**), suggesting that Omicron XBB.1.16 infection can enhance humoral immunity. However, as compared to XBB.1.16 uninfected individuals, XBB.1.16 infection did not further enhance the specific anti-XBB.1.16 Nab (**Figure 6D**). The 21 individuals who suffered from XBB.1.16 infection were classified into HLA-B^*^15 and non-HLA-B^*^15 groups, ELISA revealed that at M1 and M3 post-BA.5.2 infection, serum from HLA-B^*^15 individuals manifested dramatically lower concentrations of cross-reactive anti-XBB.1.16 Nab than did serum from non-HLA-B^*^15 participants (others) (**Figure 6E**), indicating that HLA-B^*^15 individuals are more susceptible to XBB.1.16 infection.

We finally monitored the efficacy of serum neutralizing to various SARS-CoV-2 strains, and sera from some volunteers (**Figure 6F**) and HLA-B^*^15 individuals (**Figure 6G**) at vax booster-M1, BA.5.2-M1, and XBB.1.16-M1 were selected. Results showed that these sera predominantly neutralized the SARS-CoV-2 prototype strain and Omicron BA.5.2, however, they displayed very low neutralizing activity against other Omicron variant strains. Importantly, XBB.1.16 infection did not further enhance the efficacy of viral neutralization.

## 2 Discussion

The COVID-19 pandemic represents one of the most serious public health problems worldwide, and SARS-CoV-2 is still continuous mutation during transmission (16, 17). Omicron variants exhibit more than 60 amino acid mutations in the spike protein, making it significantly distinct from the original prototype strain (18), therefore, high mutability increases the susceptibility of hosts to recurrent Omicron variant infections. Currently, the Ad26.COV2.S, mRNA-1273, the Pfizer BioNTech-Comirnaty vaccine, and the Sinovac-CoronaVac vaccine have been listed as qualified vaccines for emergency use by WHO (5). However, individuals who were received these vaccines do not effectively prevent SARS-CoV-2 variant infections, although COVID-19-related hospitalization, severity, and mortality are reduced markedly (19). In China, the most commonly used vaccines include BBIBP-CorV, CoronaVac, and Ad5-nCoV, and preliminary investigations indicate that Ad5-nCoV vaccine can trigger robust humoral and cellular immunity against SARS-CoV-2 within 28 days, and the Nab levels were declined significantly after 6 months (20). Recipients administered the CoronaVac vaccine also exhibited a notable increase in anti-SARS-CoV-2 prototype Nab levels (21). Similar to these observations, we here illustrated that individuals vaccinated with booster dose of the Ad5-nCoV and the CoronaVac manifested high levels of anti-SARS-CoV-2 prototype Nab (**Figures 2A, B**), however, the Ad5-nCoV vaccine seems to fail to elicit specific cross-reactive anti-BA.5.2 Nab (**Figure 4A**) and XBB.1.16-Nab (**Figure 6B**), indicating that the Ad5-nCoV vaccine may offer limited effectiveness in preventing Omicron variant breakthrough infection.

Early studies have highlighted the associations between certain HLA expression and the rapid dissemination of HIV, HBV, HCV, and SARS-CoV (22). Recently, HLA molecules have also been described as crucial factor in determining the outcome of SARS-CoV-2 infection and vaccination efficacy. For example, the HLA-DRB1^*^04 may predict disease severity in Iranian COVID-19 patients (23). The specific HLA supertypes, including the HLA-DRB1^*^15:01, DQB1^*^06:02, B^*^27:07, HLA-A^*^03:01, A^*^11:01, A^*^24:02, B^*^52:01, and C^*^12:02, are significantly correlated with severe/critical illness following SARS-CoV-2 infection (8, 9). Some Omicron sublineages can downregulate HLA antigens in virus-infected cells through enhancing autophagy and antagonizing CTL-induced cell death (24). Furthermore, certain HLA supertypes, such as the DQA1^*^03:03, DQB1^*^06, DRB1^*^03:01, and DRB1^*^07:01, augment vaccine-induced anti-SARS-CoV-2 Nab levels (10, 25). Furthermore, it seems that HLA-II molecules, such as the DRB1^*^03:01, DRB1^*^07:01, and DRB1^*^12:01, successfully induce Nab to counteract SARS-CoV-2 in recipients after they were administration of the COVID-19 mRNA vaccine (26). Conversely, some studies revealed a negative association between specific HLA supertypes and vaccine efficacy (27). We here confirmed a significantly positive correlation between HLA-B^*^15 alleles and seral titers of anti-prototype Nab elicited by the Ad5-nCoV vaccine and the CoronaVac-inactivated vaccine (**Figure 2**). Previous investigations have demonstrated that individuals carrying HLA-B^*^15:01 tend to experience asymptomatic SARS-CoV-2 infections (25, 28–30). Remarkably, we here showed that the HLA-B^*^15 patients experienced notably more severe clinical manifestations than that in the non-HLA-B^*^15 persons (**Figure 3E**). Most importantly, HLA-B^*^15 individuals exhibited markedly lower levels of cross-reactive specific anti-prototype Nab, including anti-BA.5.2 and anti-XBB.1.16 Nab, thus leading to diminished virus neutralization capacity against the Omicron variants. We therefore concluded that HLA-B^*^15 populations have a better response to SARS-CoV-2 vaccines but are more susceptible to Omicron breakthrough infection.

Recent studies have suggested that “Original antigenic sin” (OAS) or “immune imprinting” are involved in mediating SARS-CoV-2 new variant breakthrough infection, and evidences are also available regarding their impact on the safety and effectiveness of COVID-19 vaccines (31). For instance, individuals previously infected with SARS-CoV-2 (Wuhan strain) did not exhibit a substantial increase in Nab and T-cell responses against Omicron B.1.1.529 upon reinfection (18). Xie X et al. demonstrated that antibodies isolated from COVID-19 patients who experienced breakthrough infections with BA.2 and BA.5 showed significantly diminished neutralizing activity and antibody diversities against other variants, such as BQ.1.1.10 (BQ.1.1 + Y144del), BA.4.6.3, XBB, and CH.1.1, due to the presence of OAS (32). Zhang Z et al. confirmed that BA.2 variant breakthrough infection successfully elicits immune memory B cells derived from the prototype strain or its close variants (including Alpha, Beta, Gamma, and Delta), while the specific antibody response against BA.2 is complicated (33). Here, we showed that the Ad5-nCoV cannot induce cross-reactive anti-BA.5.2 and anti-XBB.1.16 Nab, interestingly, both Nabs were triggered by post-BA.5.2 infection. Additionally, secondary XBB.1.16 infection further enhanced anti-XBB.1.16 Nab, suggesting that immune imprinting is not obviously presented in these volunteers. However, there was a greater reduction in the number of CD19^+^CD27^+^ memory B cells in HLA-B^*^15 individuals than that in other individuals after BA.5.2 infection, this phenomenon might be responsible for the lower level of anti-prototype, anti-BA.5.2 and anti-XBB.1.16 Nab release after BA.5.2 infection.

CD4^+^ follicular helper T (Tfh) cells play pivotal roles in regulating antibody responses and maintaining long-term memory B cells (34). It seems that the induction of antigen-specific germinal center formation and the production of Nab by SARS-CoV-2 mRNA vaccines are closely associated with the presence of Tfh cells *in vivo* (35). Specifically, the HLA-DPB1^*^04-restricted S_167-180_ epitope has been shown to stimulate Tfh differentiation, leading to a sustained and robust response to COVID-19 mRNA vaccines (36). Here, we revealed a significant decrease in the frequency of memory CD19^+^ CD27^+^RBD^+^ B cells postvaccination and after BA.5.2 infection (**Figure 5**), potentially resulting in decreased serum antibodies. However, whether Tfh cells also regulate memory B cells in the HLA-B*15 population need further investigation.

An effective vaccine is expected to induce both protective cellular and humoral immunity to all kinds of SARS-CoV-2 variants, and individuals vaccinated with these vaccines also can maintain long-lasting memory B and T-cells *in vivo* (37, 38). It seems that the HLA restricted CD4^+^ as well as CD8^+^ T-cell epitopes are comprehensively distributed in the whole SARS-CoV-2 proteome (39), therefore, the induction of broadly protective cellular responses is a feasible strategy to enhance the effectiveness of COVID-19 vaccines. Recently, Tai W et al., developed a lipid nanoparticle (LNP)-formulated mRNA-based T-cell-inducing antigen, which targeted three SARS-CoV-2 proteome regions that enriched human HLA-I epitopes (HLA-EPs), the sequences of HLA-EPs are highly conserved among SARS-CoV-2 variants, therefore, HLA-EPs induces broad cellular immunity and prevent SARS-CoV-2 infection (40), HLA-EPs would be one of new generations of COVID-19 vaccine, possibly can provide protection for individuals with HLA-B^*^15.

Some limitations of our study need to be noted. First, we focused only on humoral immune responses and did not explore the potential of T-cell immunity in controlling viral infection. Second, the molecular mechanisms underlying the reduction in memory B cells in HLA-B^*^15 volunteers remain unclear. Third, the potential alterations in the diversity of B-cell receptors (BCRs) within the same volunteer across different time periods and their correlation with antibody diversity remain unexplored.

Overall, we showed that individuals carrying the HLA-B^*^15 allele exhibit a more favorable response to vaccines but are more susceptible to breakthrough infections caused by Omicron variants such as BA.5.2 and XBB.1.16. Therefore, novel vaccines and immunological control strategies for this population are necessary for future COVID-19 interventions.

## 3 Materials and methods

### 3.1 Volunteer recruitment

A total of 252 volunteers were recruited for this study. Among them, 183 volunteers received the Ad5-nCoV adenovirus vector vaccine, while 69 volunteers were administered the Sinovac CoronaVac inactivated vaccine. The ages of the volunteers were 18~59 years, and they were not pregnant and had no communicable diseases such as malignancies, diabetes, coronary heart disease, chronic obstructive pulmonary disease, stroke, chronic kidney disease, or chronic infectious diseases such as HIV, HBV, or tuberculosis. Blood samples were collected at various time points, including before the vax booster (Pre) and at 1 month (M1), 3 months (M3) and 6 months (M6) after the vax booster. Additionally, samples from Ad5-nCoV-vaccinated volunteers were collected at 1 month (M1), 3 months (M3) and 6 months (M6) after recovery from BA.5.2 breakthrough infection. Furthermore, blood samples from 21 individuals who recovered from secondary XBB.1.16 breakthrough infection at 1 month (M1) were also collected. Relevant experiments regarding vaccinated individuals were approved by the Ethics Committee of the First Affiliated Hospital of Guangzhou Medical University (2021-78).

### 3.2 Bulk HLA-ABC typing

Total RNA from PBMCs was extracted and converted into full-length cDNA using high-efficiency reverse transcriptase. The targeted genes were captured using a highly specific capture panel, and the resulting products were supplemented with sequencing primers to generate libraries suitable for sequencing. Following library construction, quantitative analysis was performed using Qubit 3.0, followed by insert size detection using Qsp100. Once the insert size met the expected criteria, paired-end 150 (PE150) sequencing was conducted using the Illumina X10 or NovaSeq 6000 platform. After obtaining the raw sequencing data, data filtering and quality control were performed using fastp (v0.21.0) software. The high-quality sequencing data obtained after filtering were then analyzed for HLA typing using arcasHLA software.

### 3.3 Detection of anti-SARS-CoV-2 spike (S1+S2) and Nab antibodies

An indirect enzyme-linked immunosorbent assay (ELISA) kit provided by Sino Biological, Inc. (Cat: KIT004) and (Cat: CSB-EL33243HU) was used to measure antibodies was used as described by the manufacturer, and the intensity of the color was measured at 450 nm.

### 3.4 Detection of anti-BA.5.2 and anti-XBB.1.16 neutralizing antibodies

An indirect enzyme-linked immunosorbent assay (ELISA) kit provided by ACROBiosystems (Cat: N107-CN.01, N173-CN.01) was used. This assay kit was used to measure the levels of anti-SARS-CoV-2 neutralizing antibody through a competitive ELISA. The microplate in the kit was precoated with human ACE2 protein. The experiment included 5 simple steps and the absorbance at 450 nm minus the absorbance at 630 nm to remove background prior to statistical analysis. The OD value reflects the amount of protein bound.

### 3.5 Pseudovirus neutralization assay

The prototype strain and SARS-CoV-2 variants were all self-prepared by Zhongke Guobang (Beijing) Inspection and Testing Co., Ltd. Pseudovirus dilution to 1.3x10^4^/mL using complete DMEM. Vero cells were prepared from the incubator, counted after trypsin digestion, and diluted to a concentration of 2x10^5^ cells/mL using complete DMEM. The plate was then agitated for 2 minutes, and the luminescence was measured using a multifunctional plate reader. Analysis software specific to neutralizing antibody data for COVID-19 was used throughout the calculation process.

### 3.6 The live virus neutralization 

Virus was titrated using a focus-forming assay (FFA). Vero E6 cells were seeded in 96-well plates one day before infection. The plasma samples were serially diluted and incubated with an equal volume of virus at 37°C for 1 h. Then, the mixtures were inoculated onto Vero E6 cells at 37°C for 1 h. The cells were then incubated with a rabbit anti-SARS-CoV-2 nucleocapsid protein polyclonal antibody (Cat. No. 40143-T62, Sino Biological, Inc., Beijing), followed by an HRP-labeled goat anti-rabbit secondary antibody (Cat. No. 109-035-088, Jackson ImmunoResearch Laboratories, Inc., West Grove, PA). The foci were visualized by TrueBlue Peroxidase Substrate (KPL, Gaithersburg, MD) and counted with an ELISPOT reader. Viral titers were calculated as the FFU per ml or per gram of tissue.

### 3.7 Flow cytometry

Peripheral blood mononuclear cells (PBMCs) (approximately 1 x 106 cells/100 µL) were collected at various time points postvaccination and postinfection. The cells were centrifuged at 1000 × g for 5 minutes at 4°C and washed twice with PBS (catalog no. 40143-T62, Gibco). Subsequently, the cells were stained with recombinant SARS-CoV-2 BA.4/BA.5 The RBD Alexa Fluor^®^ 488 protein dye (Catalog No.: AFG11229-020, R&D), recombinant SARS-CoV-2 spike RBD Alexa Fluor^®^ 647 protein (Catalog No.: AFR10500-020, R&D), CD38-Brilliant Violet 421 (Catalog No.: 397119, BioLegend), and CD20-PE/Cyanine7 (Catalog No.: 356617, BioLegend) were diluted (1:100) in FACs buffer (0.1% BSA) at 4°C for 30 minutes. After staining, the cells were washed twice with FACs staining buffer (0.1% BSA). After three washes, the cells were resuspended buffer and filtered through a 35 µm filter. Cell collection was performed using BD FACSaria III, and analysis was conducted using FlowJo 10.8.1.

### 3.8 Statistical analysis

The data were entered into Excel, and statistical analysis and graphical representations were performed by R-4.3.3. Statistical analysis between two samples was conducted using a t test, while analysis involving three samples or unpaired comparisons was conducted using the Wilcoxon test. Pairwise comparisons among three or more samples were conducted using Tukey’s multiple comparisons test. All the statistical tests were two-sided, and a p value less than 0.05 indicated statistical significance.

## Data Availability

The raw sequence data can be obtained from the GSA-Human database (https://ngdc.cncb.ac.cn/gsa-human) with accession number HRA008235. The code that supports the findings of this study is available from the corresponding authors upon request.

## References

[1] W.c.C.-. dashboard. (2024). Available online at: https://covid19.who.int/.

[2] Zhang Y Zhang HC Zhang WH. SARS-CoV-2 variants, immune escape, and countermeasures. Front Med-Prc. (2022) 16:196–207. doi: 10.1007/s11684-021-0906-x PMC8898658 35253097

[3] Chen KWK Huang DTN Huang LM. SARS-CoV-2 variants- Evolution, spike protein, and vaccines. BioMed J. (2022) 45:573–9. doi: 10.1016/j.bj.2022.04.006 PMC9072773 35526825

[4] Fiolet T Kherabi Y MacDonald CJ Ghosn J Peiffer-Smadja N. Comparing COVID-19 vaccines for their characteristics, efficacy and effectiveness against SARS-CoV-2 and variants of concern: a narrative review. Clin Microbiol Infect. (2022) 28:202–21. doi: 10.1016/j.cmi.2021.10.005 PMC8548286 34715347

[5] Hassine IH. Covid-19 vaccines and variants of concern: A review. Rev Med Virol. (2022) 32:e2313. doi: 10.1002/rmv.2313 34755408 PMC8646685

[6] Liu JY Chandrashekar A Sellers D Barrett J Jacob-Dolan C Lifton M. Vaccines elicit highly conserved cellular immunity to SARS-CoV-2 Omicron. Nature. (2022) 603:493–+. doi: 10.1038/s41586-022-04465-y PMC8930761 35102312

[7] Gao Y Cai C Grifoni A Muller TR Niessl J Olofsson A. Ancestral SARS-CoV-2-specific T cells cross-recognize the Omicron variant. Nat Med. (2022) 28:472–+. doi: 10.1038/s41591-022-01700-x PMC8938268 35042228

[8] Novelli A Andreani M Biancolella M Liberatoscioli L Passarelli C Colona VL. HLA allele frequencies and susceptibility to COVID-19 in a group of 99 Italian patients. Hla. (2020) 96:610–4. doi: 10.1111/tan.14047 PMC7461491 32827207

[9] Ng MHL Lau KM Li L Cheng SH Chan WY Hui PK. Association of human-leukocyte-antigen class I (B*0703) and class II (DRB1*0301) genotypes with susceptibility and resistance to the development of severe acute respiratory syndrome. J Infect Dis. (2004) 190:515–8. doi: 10.1086/421523 PMC7109646 15243926

[10] Khor SS Omae Y Takeuchi JS Fukunaga A Yamamoto S Tanaka A. An association study of HLA with the kinetics of SARS-CoV-2 spike specific IgG antibody responses to BNT162b2 mRNA vaccine. Vaccines-Basel. (2022) 10:563. doi: 10.3390/vaccines10040563 35455312 PMC9029840

[11] Astbury S Reynolds CJ Butler DK Munoz-Sandoval DC Lin KM Pieper FP. HLA-DR polymorphism in SARS-CoV-2 infection and susceptibility to symptomatic COVID-19. Immunology. (2022) 166:68–77. doi: 10.1111/imm.13450 35156709 PMC9111350

[12] Weidner L Kalser J Kreil TR Jungbauer C Mayr WR. Neutralizing antibodies against SARS-CoV-2 and HLA class I and II polymorphism. Transfus Med Hemoth. (2021) 48:173–4. doi: 10.1159/000515149 PMC8018198 34177422

[13] Guzmán-López S Darwich-Salazar A Bocanegra-Ibarias P Salas-Treviño D Flores-Treviño S Pérez-Alba E. Clinical and immunologic efficacy of the recombinant adenovirus type-5-vectored (CanSino bio) vaccine in university professors during the COVID-19 delta wave. Vaccines-Basel. (2022) 10:656. doi: 10.3390/vaccines10050656 35632412 PMC9143224

[14] Richardson VL Franco MAC Márquez AB Valdez LM Ceronio LEC Cruz VC. Vaccine effectiveness of CanSino (Adv5-nCoV) coronavirus disease 2019 (COVID-19) vaccine among childcare workers-Mexico, March-December 2021. Clin Infect Dis. (2022) 75:S167–73. doi: 10.1093/cid/ciac488 PMC9214173 35717650

[15] Wolday D Fung CYJ Morgan G Casalino S Frangione E Taher J. HLA variation and SARS-CoV-2 specific antibody response. Viruses-Basel. (2023) 15:906. doi: 10.3390/v15040906 PMC10143129 37112884

[16] Fernandes Q Inchakalody VP Merhi M Mestiri S Taib N El-Ella DMA. Emerging COVID-19 variants and their impact on SARS-CoV-2 diagnosis, therapeutics and vaccines. Ann Med. (2022) 54:524–40. doi: 10.1080/07853890.2022.2031274 PMC8843115 35132910

[17] Umakanthan S Sahu P Ranade AV Bukelo MM Rao JS Abrahao-MaChado LF. Origin, transmission, diagnosis and management of coronavirus disease 2019 (COVID-19). Postgrad Med J. (2020) 96:753–8. doi: 10.1136/postgradmedj-2020-138234 PMC10016932 32563999

[18] Addetia A Piccoli L Case JB Park YJ Beltramello M Guarino B. Neutralization, effector function and immune imprinting of Omicron variants. Nature. (2023) 621:592–+. doi: 10.1038/s41586-023-06487-6 PMC10511321 37648855

[19] Zeng BQ Gao L Zhou QX Yu K Sun F. Effectiveness of COVID-19 vaccines against SARS-CoV-2 variants of concern: a systematic review and meta-analysis. BMC Med. (2022) 20:200. doi: 10.1186/s12916-022-02397-y 35606843 PMC9126103

[20] Zhu FC Li YH Guan XH Hou LH Wang WJ Li JX. Safety, tolerability, and immunogenicity of a recombinant adenovirus type-5 vectored COVID-19 vaccine: a dose-escalation, open-label, non-randomised, first-in-human trial. Lancet. (2020) 395:1845–54. doi: 10.1016/S0140-6736(20)31208-3 PMC7255193 32450106

[21] Wu ZW Hu YL Xu M Chen Z Yang WQ Jiang ZW. Safety, tolerability, and immunogenicity of an inactivated SARS-CoV-2 vaccine (CoronaVac) in healthy adults aged 60 years and older: a randomised, double-blind, placebo-controlled, phase 1/2 clinical trial. Lancet Infect Dis. (2021) 21:803–12. doi: 10.1016/S1473-3099(20)30987-7 PMC7906628 33548194

[22] Blackwell JM Jamieson SE Burgner D. HLA and infectious diseases. Clin Microbiol Rev. (2009) 22:370–+. doi: 10.1128/CMR.00048-08 PMC2668228 19366919

[23] Ebrahimi S Ghasemi-Basir HR Majzoobi MM Rasouli-Saravani A Hajilooi M Solgi G. HLA-DRB1*04 may predict the severity of disease in a group of Iranian COVID-19 patients. Hum Immunol. (2021) 82:719–25. doi: 10.1016/j.humimm.2021.07.004 PMC8275473 34294460

[24] Moriyama M Lucas C Monteiro VS Iwasaki A Surveile YSCG. Enhanced inhibition of MHC-I expression by SARS-CoV-2 Omicron subvariants. P Natl Acad Sci U S A. (2023) 120:e2221652120. doi: 10.1073/pnas.2221652120 PMC10120007 37036977

[25] Higuchi T Oka S Furukawa H Tohma S. Associations of polymorphisms with anti-SARS-CoV-2 spike and neutralizing antibody titers in Japanese rheumatoid arthritis patients vaccinated with BNT162b2. Vaccines-Basel. (2023) 11:404. doi: 10.3390/vaccines11020404 36851281 PMC9965868

[26] Huang CQ Vishwanath S Carnell GW Chan ACY Heeney JL. Immune imprinting and next-generation coronavirus vaccines. Nat Microbiol. (2023) 8:1971–85. doi: 10.1038/s41564-023-01505-9 37932355

[27] Gerhards C Kittel M Ast V Bugert P Froelich MF Hetjens M. Humoral SARS-CoV-2 immune response in COVID-19 recovered vaccinated and unvaccinated individuals related to post-COVID-syndrome. Viruses-Basel. (2023) 15:454. doi: 10.3390/v15020454 PMC9966735 36851668

[28] Triunfol M. HLA-B*15:01 allele and asymptomatic SARS-CoV-2 infection. Lancet Resp Med. (2023) 11:E83–3. doi: 10.1016/S2213-2600(23)00295-3 37549678

[29] Bordon Y. Asymptomatic SARS-CoV-2 infections linked to HLA-B*15:01. Nat Rev Genet. (2023) 24:663–3. doi: 10.1038/s41576-023-00641-6 37507492

[30] Augusto DG Murdolo LD Chatzileontiadou DSM Sabatino JJ Yusufali T Peyser ND. A common allele of is associated with asymptomatic SARS-CoV-2 infection. Nature. (2023) 620:128–36. doi: 10.1038/s41586-023-06331-x PMC10396966 37468623

[31] Aguilar-Bretones M Fouchier RAM Koopmans MPG van Nierop GP. Impact of antigenic evolution and original antigenic sin on SARS-CoV-2 immunity. J Clin Invest. (2023) 133:e162192. doi: 10.1172/JCI162192 36594464 PMC9797340

[32] Cao YL Jian FC Wang J Yu YL Song WL Yisimayi A. Imprinted SARS-CoV-2 humoral immunity induces convergent Omicron RBD evolution. Nature. (2023) 614:521–9. doi: 10.1038/s41586-022-05644-7 PMC9931576 36535326

[33] Ju B Fan Q Wang M Liao XJ Guo HM Wang HY. Antigenic sin of wild-type SARS-CoV-2 vaccine shapes poor cross-neutralization of BA.4/5/2.75 subvariants in BA.2 breakthrough infections. Nat Commun. (2022) 13:7120. doi: 10.1038/s41467-022-34400-8 36402756 PMC9675777

[34] Zhang J Wu Q Liu ZY Wang QJ Wu JJ Hu YB. Spike-specific circulating T follicular helper cell and cross-neutralizing antibody responses in COVID-19-convalescent individuals. Nat Microbiol. (2021) 6:51–+. doi: 10.1038/s41564-020-00824-5 33199863

[35] Lederer K Castaño D Atria DG Oguin TH Wang S Manzoni TB. SARS-CoV-2 mRNA vaccines foster potent antigen-specific germinal center responses associated with neutralizing antibody generation. Immunity. (2020) 53:1281–+. doi: 10.1016/j.immuni.2020.11.009 PMC7680029 33296685

[36] Mudd PA Minervina AA Pogorelyy MV Turner JS Kim W Kalaidina E. SARS-CoV-2 mRNA vaccination elicits a robust and persistent T follicular helper cell response in humans. Cell. (2022) 185:603–+. doi: 10.1016/j.cell.2021.12.026 PMC8695127 35026152

[37] Moderbacher CR Ramirez S Dan JM Grifoni A Hastie KM Weiskopf D. Antigen-specific adaptive immunity to SARS-CoV-2 in acute COVID-19 and associations with age and disease severity. Cell. (2020) 183:996–+. doi: 10.1016/j.cell.2020.09.038 PMC7494270 33010815

[38] Sette A Crotty S. Adaptive immunity to SARS-Cov-2 and COVID-19. Cell. (2021) 184:861–80. doi: 10.1016/j.cell.2021.01.007 PMC7803150 33497610

[39] Peng YC Mentzer AJ Liu GH Yao X Yin ZX Dong DN. Broad and strong memory CD4 and CD8 T cells induced by SARS-CoV-2 in UK convalescent individuals following COVID-19. Nat Immunol. (2020) 21:1336–+. doi: 10.1038/s41590-020-0782-6 PMC7611020 32887977

[40] Tai WB Feng SY Chai BJ Lu SY Zhao GY Chen D. An mRNA-based T-cell-inducing antigen strengthens COVID-19 vaccine against SARS-CoV-2 variants. Nat Commun. (2023) 14:2962. doi: 10.1038/s41467-023-38751-8 37221158 PMC10204679

