# Supplementary material for: Individuals carrying the HLA-B*15 allele exhibit favorable responses to COVID-19 vaccines but are more susceptible to Omicron BA.5.2 and XBB.1.16 infection
Source: Front Immunol. 2024 Aug 27;15:1440819. doi: 10.3389/fimmu.2024.1440819 (PMC11383769; doi:10.3389/fimmu.2024.1440819)
Supplement: Supplementary file 1 [file DataSheet1.pdf]

Supplementary figures and figure legend

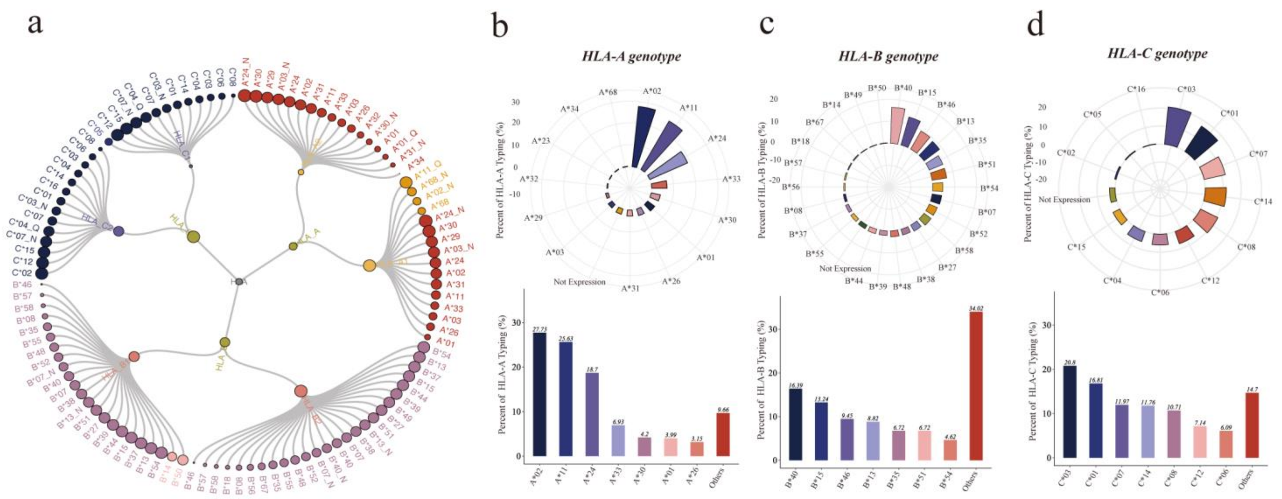

sFig.1 the HLA-ABC genotype polymorphisms was analzyed with utilizing the MiSeqDx™ Next Generation Sequencing (NGS) platform. (**a**) A total of 233 volunteers (Ad5-nCoV, n = 168; CoronaVac n = 65) were recruited to detect the HLA-ABC genotype polymorphisms. Results showed that the (**b**) HLA-A, (c) HLA-B, and (d) HLA-C genotypes of these volunteers.

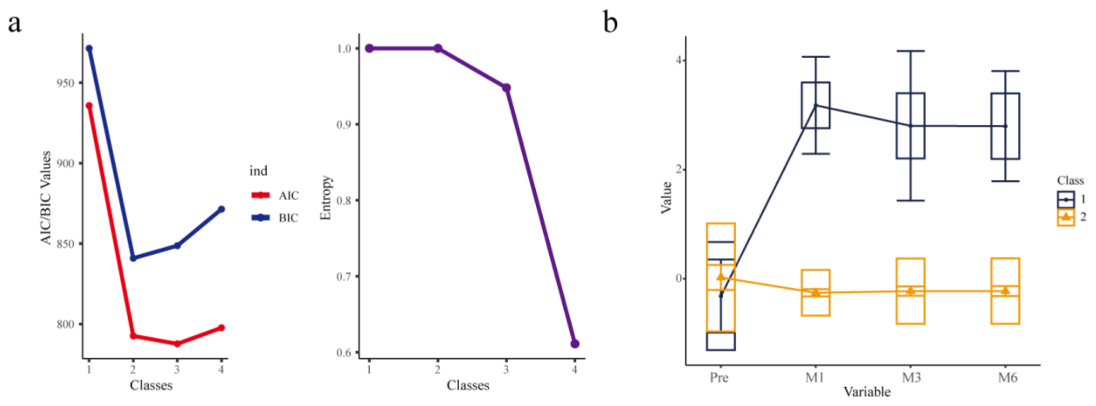

sFig.2 LPA was used to establish the latent profile model. Based on the 1 classification model, the number of profiles was gradually increased to explore the potential profiles of 1 to 4 classifications, and the fitness fitting index of each model was calculated. (**a**) Smaller values of Akaike information criterion (AIC) and Bayesian information criterion (BIC) indicate a better fit of the model. Upon the Entropy value reaches 0.80 or above, the accuracy of classification is higher, and the closer to 1, the higher the credibility of classification. (**b**) Classification results of the LPA model for 94 people vaccinated with adenovirus vaccine when the number of classifications was equal to 2.

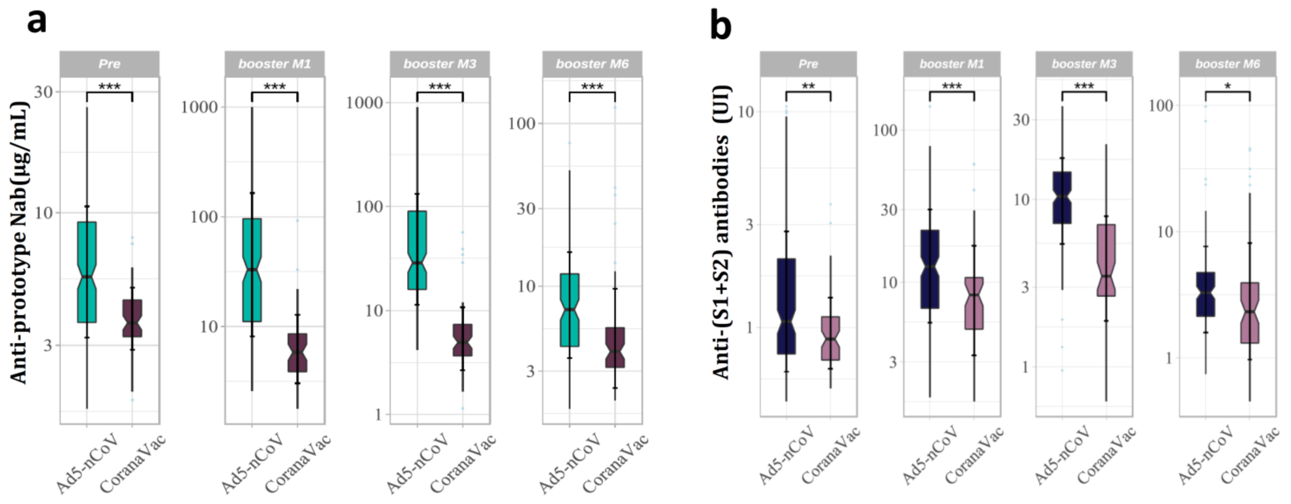

sFig.3 the efficacy of the Ad5-nCoV vaccine is better than the CoronaVac inactivated vaccine. (**a**) The concentrations of both SARS-CoV-2 specific anti-prototype Nab, and (**b**) anti-(S1+S2) antibodies, were compared side by side. $^{*}p$<0.05, and $^{***}p$<0.0001.

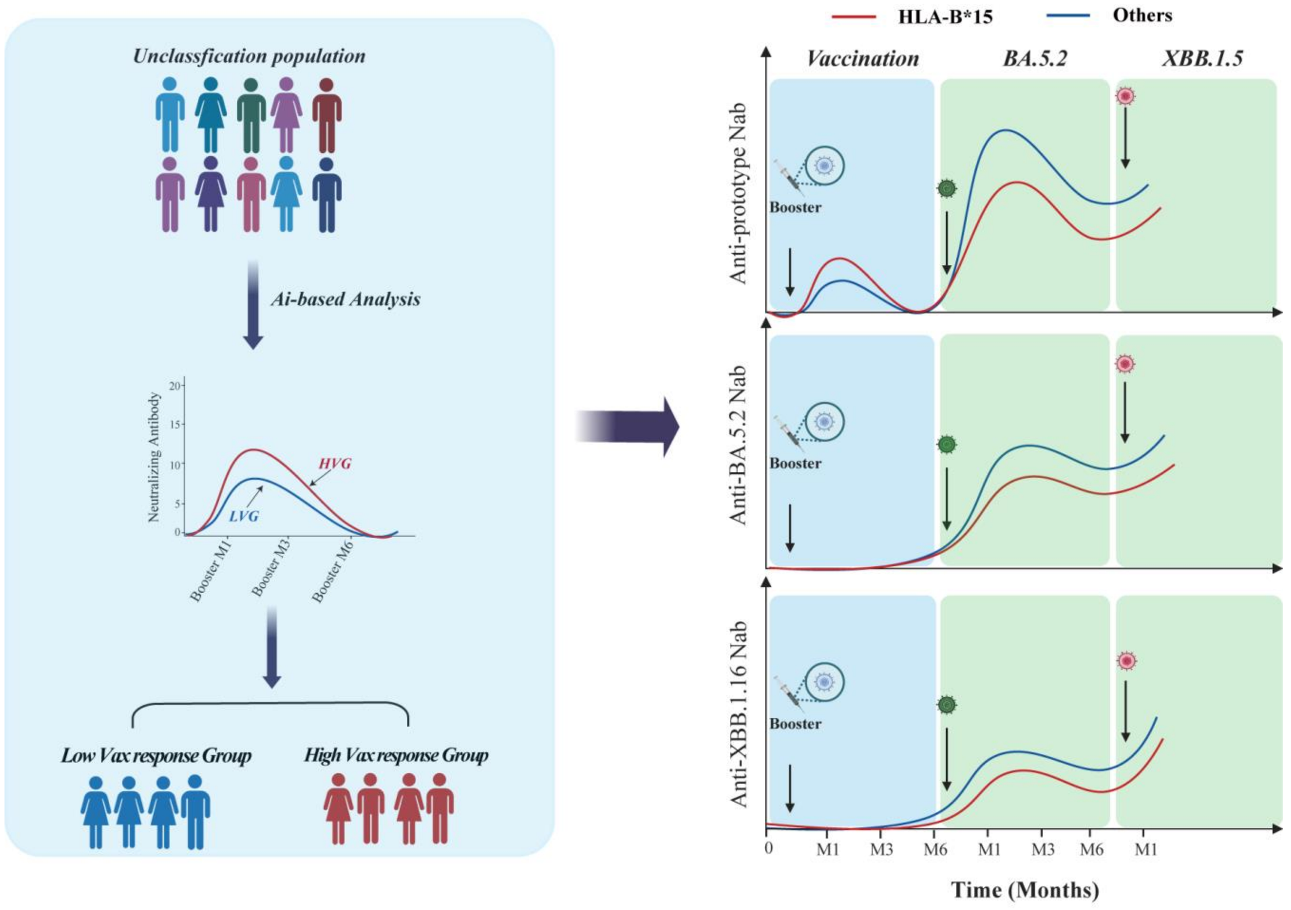

sFig.4 The "Graphical Abstracts" about this study. The vax volunteers could be divided into two groups (low vax response group and high vax response group) based on anti-prototype Nab. Interestingly, we found the HLA-B*15 volunteers exhibit a favorable response to COVID-19 vaccines but are more susceptible to Omicron BA.5.2 and XBB.1.16 infections due to they possess poorer levels of anti-BA.5.2 and anti-XBB.1.16 Nab.
